# Revealing the Key MSCs Niches and Pathogenic Genes in Influencing CEP Homeostasis: A Conjoint Analysis of Single-Cell and WGCNA

**DOI:** 10.3389/fimmu.2022.933721

**Published:** 2022-06-27

**Authors:** Weihang Li, Shilei Zhang, Yingjing Zhao, Dong Wang, Quan Shi, Ziyi Ding, Yongchun Wang, Bo Gao, Ming Yan

**Affiliations:** ^1^ Department of Orthopedic Surgery, Xijing Hospital, Air Force Medical University, Xi’an, China; ^2^ Department of Intensive Care Unit, Nanjing First Hospital, Nanjing Medical University, Nanjing, China; ^3^ Department of Orthopaedics, Affiliated Hospital of Yanan University, Yanan, China; ^4^ Department of Aerospace Medical Training, School of Aerospace Medicine, Air Force Medical University, Xi’an, China; ^5^ Key Lab of Aerospace Medicine, Chinese Ministry of Education, Xi’an, China

**Keywords:** degenerative disc disease, cartilage endplate, multipotent stem cells, immune infiltration, WGCNA, single-cell transcriptomic landscape construction

## Abstract

Degenerative disc disease (DDD), a major contributor to discogenic pain, which is mainly resulted from the dysfunction of nucleus pulposus (NP), annulus fibrosis (AF) and cartilage endplate (CEP) cells. Genetic and cellular components alterations in CEP may influence disc homeostasis, while few single-cell RNA sequencing (scRNA-seq) report in CEP makes it a challenge to evaluate cellular heterogeneity in CEP. Here, this study conducted a first conjoint analysis of weighted gene co-expression network analysis (WGCNA) and scRNA-seq in CEP, systematically analyzed the interested module, immune infiltration situation, and cell niches in CEP. WGCNA and protein-protein interaction (PPI) network determined a group of gene signatures responsible for degenerative CEP, including BRD4, RAF1, ANGPT1, CHD7 and NOP56; differentially immune analysis elucidated that CD4^+^ T cells, NK cells and dendritic cells were highly activated in degenerative CEP; then single-cell resolution transcriptomic landscape further identified several mesenchymal stem cells and other cellular components focused on human CEP, which illuminated niche atlas of different cell subpopulations: 8 populations were identified by distinct molecular signatures. Among which, NP progenitor/mesenchymal stem cells (NPMSC), also served as multipotent stem cells in CEP, exhibited regenerative and therapeutic potentials in promoting bone repair and maintaining bone homeostasis through SPP1, NRP1-related cascade reactions; regulatory and effector mesenchymal chondrocytes could be further classified into 2 different subtypes, and each subtype behaved potential opposite effects in maintaining cartilage homeostasis; next, the potential functional differences of each mesenchymal stem cell populations and the possible interactions with different cell types analysis revealed that JAG1, SPP1, MIF and PDGF etc. generated by different cells could regulate the CEP homeostasis by bone formation or angiogenesis, which could be served as novel therapeutic targets for degenerative CEP. In brief, this study mainly revealed the mesenchymal stem cells populations complexity and phenotypic characteristics in CEP. In brief, this study filled the gap in the knowledge of CEP components, further enhanced researchers’ understanding of CEP and their cell niches constitution.

## Introduction

Degenerative disc disease (DDD), regarded as the most common trigger of back pain, is a widespread global healthcare disease and has resulted in a significant socioeconomic burden consistently (1). DDD is a chronic and progressive disease, characterized by various clinical symptoms, including weakness of extremities, paresthesia and back pain (2–4). The current main treatments regarding DDD contain conservative approaches like bed rest, analgesic drugs, rehabilitation, etc., and non-conservative like interventional and surgical therapy, while these treatments are only limited to provide symptomatic reliefs but fail to restore the homeostasis of intervertebral disc (IVD) among body system (5). Therefore, the relentless threat of DDD to human health and social economics all motivate more researches and understandings of the physiology and pathology of IVD.

IVD located between adjacent vertebrae and act as fibrocartilaginous tissues to provide flexibility, loading support, energy storage and consumption in the spine (6). As the largest avascular organ in body, IVD is comprised of three special parts: central nucleus pulposus (NPs), surrounding lamellar annulus fibrosis (AFs) and upper and lower cartilage endplates (CEPs) which is adjoin to the vertebra (7). This special structure makes it immune-privilege organ that prevents vascular infiltration. While DDD may occur when the homeostatic states in IVD are destroyed, such as decomposition of extracellular matrix (ECM), decrease of NP and AF, ingrowth of vessels, and cellular or biochemical alterations of microenvironment in IVD (8, 9).

The etiology of DDD is mainly blamed of genetic change, accounting for more than 70% (10, 11). Most of the studies have mainly reported the researches and applications of NP and AF in the development of DDD, including pathogenic mechanisms, therapies or diagnosis (12–14), some therapeutic approaches provided a promising prospect in the treatment of disc degeneration, like natural drugs, exosomes (14, 15). Gao et al. reported the first map for the genetic mice model based on purified NPs, which revealed the heterogeneity and diverse roles for NP cell populations during homeostasis and degeneration of disc (16); And also, Li et al. analyzed the roles of lipid-metabolism genes in the development of disc degeneration, indicating several lipid-metabolism genes could affect disc degeneration by promote immune infiltration (17). However, researches focused on CEP were far less than NP or AF, due to the unimpressive cartilaginous/bony structure, the relationships between CEP and DDD remained poorly understood, thus, the detailed underlying mechanisms of degenerative CEP is worth discussing.

**Figure 1:**
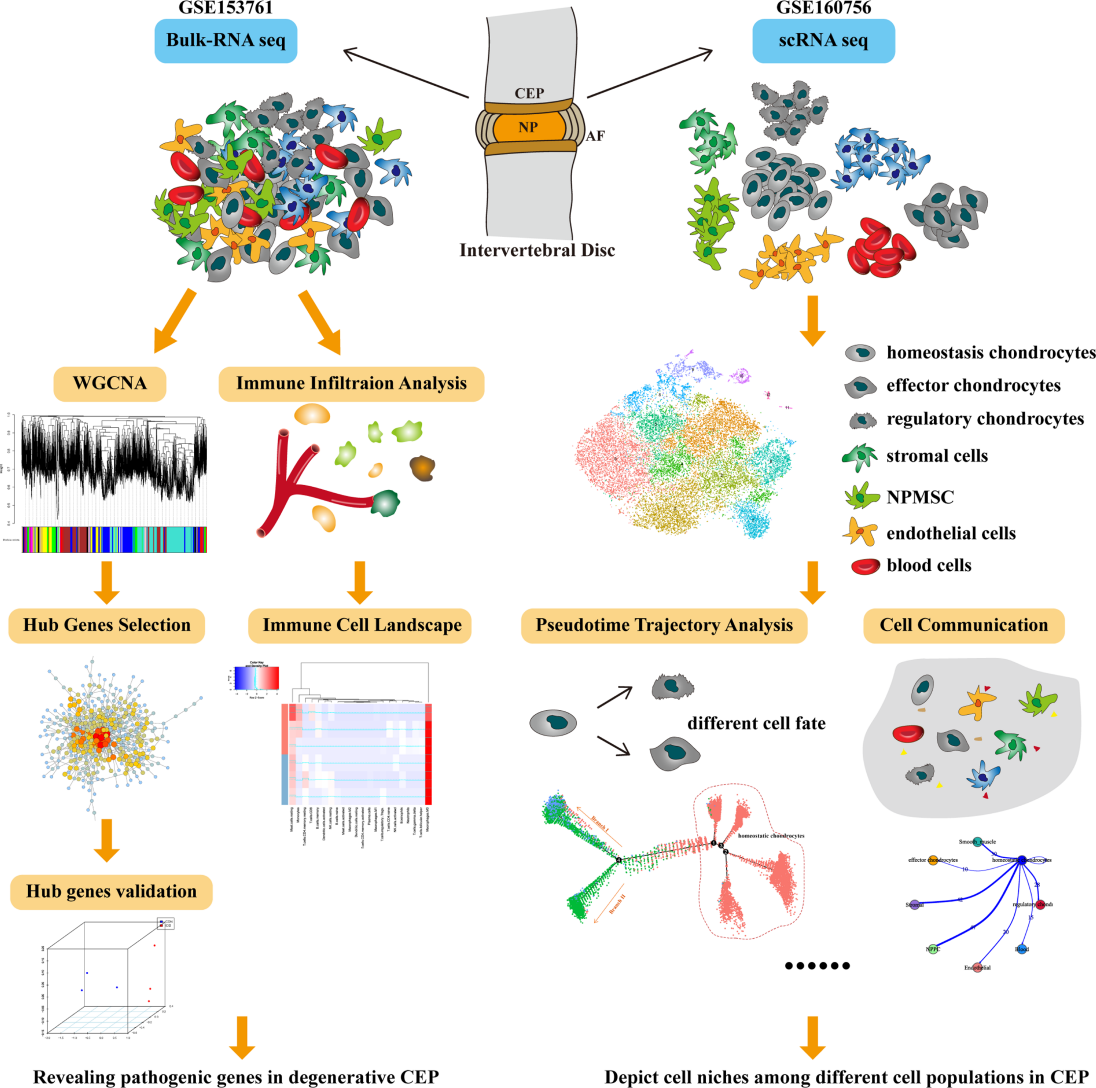
The whole diagram and workflow of this study.

WGCNA (weighted gene co-expression network analysis) is a holistic and systematic biologic tool, aiming to explore the correlations between genes and given features through constructing gene expression matrix-based network; basically, WGCNA builds a bridge between sample characteristics and gene expression profiles (18). Single-cell RNA sequencing (scRNA-seq), served as the most important methodology progress and breakthrough technology, which could provide powerful algorithm to study the cellular heterogeneity of different tissues (19). Consequently, identifying functions and niches of CEP among IVD can further increase our understanding of spinal homeostasis and eventually provide new therapeutic approaches of DDD. In this study, we performed a first conjoint analysis of WGCNA and scRNA-seq analysis to produce cell atlas through CEP tissue, aiming to figure out the underlying pathologic mechanisms of degenerative CEP. We also systematically revealed cell-cell communications and signaling pathways engaged in the niche regulations of CEP, and elucidated the spatially regulated heterogeneity of CEPs as well as key signals that underlies homeostasis. The whole diagram and framework of this study was displayed in **Figure 1**.

## Materials and Methods

### Data Acquisition and Preprocessing

Microarray datasets were screened and downloaded from GEO (gene expression omnibus, https://www.ncbi.nlm.nih.gov/geo/) database. Among them, the gene expression matrix of CEP: GSE153761 series (series matrix file) were downloaded for WGCNA analysis, which contained 3 normal and 3 degenerative CEPs; GSE160756 data (‘.loom’ file format) were downloaded for scRNA-seq analysis, which contained NP, AF and CEP tissues, and only CEP tissues were extracted for subsequent analysis. Based on manufacture-provided probe ID-gene symbol annotation files from GPL22120 platform, corresponding probes were applied to annotate into gene symbol, probes without annotations were removed.

### Weighted Gene Co-Expression Network Analysis

All genes in GSE153761 were included for WGCNA analysis (18) (‘WGCNA’ package in R, version 4.1.2), to identify the interested gene sets, to ensure that all genes information were included. Hierarchical clustering analysis was performed to check the heterogeneity of samples and eliminate the outliers from research. Then soft threshold power was calculated to select the most optimal value for subsequent network construction, to get the real biological network state (scale-free network). The cutoff of optimal soft threshold power was set as R^2^ = 0.90 and mean connectivity = 0.

Next, weighted gene co-expression network was constructed based on the most optimal value and all genes expression. Co-expression modules were identified and clustered with each other according to the similarity of each module. The minimum number of genes in each module were set as 30 and max block size were set as 5000. Eigengenes adjacency was calculated to assess interaction of different gene modules. The degrees of correlation between genes were calculated through topological overlap measure (TOM) (20, 21).

Then module-trait Pearson’s correlations were calculated to display the relationships between module eigengene and phenotype, P < 0.01 was regarded as statistically significant. Based on the above analysis, the module with the highest correlations and lowest P values between modules and clinical samples (DDD) were visualized by GS-MM (Gene significance vs. Module membership) plot. Ultimately, this module was selected as the interested module and applied for following analysis.

### Protein-Protein Interaction Network Construction and Hub Genes Selection

Protein-protein interaction (PPI) analysis was conducted by STRING repository (Search tool for retrieval of interacting genes, https://string-db.org/), based on interested gene module screened from WGCNA. The PPI network was constructed in STRING database and then uploaded into Cytoscape software (version 3.8.0) to further screen hub modules and genes. MCODE algorithm was used for clustering given network links based on topology to identify densely connected subgroups. MCODE scores and P values were calculated and fully assessed to screen the most correlated subgroup; ‘Cytohubba’ algorithm provided 11 different topological methods including ‘Betweenness’, ‘BottleNeck’, ‘Closeness’, ‘Degree’, ‘DMNC’, ‘EcCentricity’, ‘EPC’, ‘MCC’, ‘MNC’, ‘Radiality’, ‘Stress’, which applied for identifying key targets from a complex network, thus, hub genes were identified and fully estimated by each algorithm in ‘cytohubba’, the most correlated genes appeared in each algorithm were finally determined as hub genes (22).

### Hub Genes Validation and Principal Component Analysis

Hub genes were also evaluated in GSE series to figure out the relationships between degenerative and normal CEP tissues. Hierarchical clustering heatmap of these hub genes were displayed to observe the distinguish ability between normal and degenerative CEP. Principal component analysis (PCA) was further conducted to reduce dimensions of these hub genes from high-dimension to PCA1, 2, and PCA3, which could help observe the distinguish ability between normal and DDD patients (‘princomp’ and ‘prcomp’ functions in R). The results were visualized by 3D scatter plot (‘scatterplot3d’, ‘rgl’ and ‘ggplot2’ packages in R).

### Assessment of Immune Cells Landscape Among CEPs

Totally 22 types of immune infiltrating cells were evaluated in CEP tissue, to get a comprehensive information about immune cells expression situation. The bulk-RNA sequence data of normal and degenerative CEP tissue from GSE153761 were collected for analysis. ‘Cibersort.R’ related code and standard immune cell expression file ‘LM22.txt’ were obtained from official website (https://cibersort.stanford.edu/), which were conducted in R. The abundance of immune cell members from mixed cell populations were assessed through gene expression profiles and correlation heatmap. Proportion situation and differentially changed immune cells among different groups were fully analyzed and visualized.

### Single Cell RNA Statistical Processing

The raw scRNA-seq data of CEPs (‘.loom’ file format) were obtained and downloaded from GSE160756, after the whole data were read and displayed in R, totally 91295 cells from 4 normal patients (Pfirrmann grade I, II) were concluded, which contained 27001 AF cells, 36352 NP cells and 27942 CEP cells. Only CEP cells were extracted from the whole cells and pooled for subsequent analysis. Filtering criteria was followed by a rigorous procedure: cells which contained more than 200 expressed genes and mitochondria UMI rate < 5% were passed the cell quality filtering, and all mitochondria genes were removed from expression matrix.

‘Seurat’ R package (23) (version 4.1.0) was used to create Seurat object from scRNA-seq data, followed by cell normalization and scale. The top 2000 variable features of each sample were analyzed after normalization. Then RPCA algorithm was selected as conjoint analysis to correct batch effects between different samples, ‘FindIntegrationAnchors’ function was used to merge sample files with common anchors among variables (dims = 1:30, k.anchors=10). It is highly recommended to use integrative non-negative matrix factorization (iNMF), Domain Adversarial and Variational Auto-Encoder (DAVAE), Variational Inference assisted Probabilistic Canonical Correlation Analysis (VIPCCA) algorithms (24–26) when processing multiple modalities or large scale of different datasets. PCA was constructed based on the scaled data with the top 2000 highly variable genes and the top 30 principles were selected for tSNE (t-distributed stochastic neighbor embedding) and UMAP (uniform manifold approximation and projection) dimensional reduction. The unsupervised cell clusters based on the top 30 PCA principles were acquired using the graph-based cluster method (resolution = 0.5). The marker genes of each cell population were calculated by ‘FindAllMarkers’ function under following criteria: Wilcox rank sum test algorithm; logfc.threshold > 0.25; P Value < 0.05; and min.pct > 0.1. To further identify the cell population in detail, the clusters of same cell type were further selected for re-tSNE analysis, graph-based clustering and marker genes analysis.

### Differentially Expressed Genes Identification

To identify Differentially expressed genes (DEGs) among different clusters or cell types generated from scRNA-seq data, the function ‘FindMarkers’ was performed under the following criteria: Wilcox rank sum test algorithm; logfc.threshold > 0.25; P Value < 0.05; min.pct > 0.1; and only.pos = TRUE.

### Cell Fate States Analysis (CytoTRACE and Pseudotime Analysis)

CytoTRACE analysis (27) is one of the computational methods for predicting the states of cell fate, a cell trajectory analysis using gene counts and expression, which could predict the relative differential states of cells based on scRNA-seq data. CytoTRACE was usually performed to predict the cell differentiation states without any prior information, which was applied for mesenchymal stem cells stage analysis with default parameter.

Pseudotime analysis (28) is another cell fate analytic method, the single-cell trajectory analysis was conducted using Monocle2 algorithm (http://cole-trapnell-lab.github.io/monocle-release), followed by DDR-Tree and default parameters. Before Monocle analysis, the Seurat clustering result and raw expression counts matrix of cells passed the filtering criteria were selected and prepared. Based on the pseudo-time trajectory, branch expression analysis modeling (BEAM analysis) was performed for branch fate-determined gene analysis.

### Cell-Cell Communication Analysis

To obtain a comprehensive view of cell-cell communication information, CellphoneDB (29) was enabled to get a systematic analysis for cell-cell communication molecules, which was a public repository of ligands, receptors and their interactions. The membrane, secreted and peripheral proteins of the clusters of different time points were annotated. Significant mean and cell communication significance (P Value < 0.05) were calculated based on the molecule interactions and the normalized gene matrix achieved by Seurat normalization.

### Functional and Pathway Enrichment Analysis

DEGs or marker genes of cell types were analyzed by R [‘clusterProfiler’ package (30)], to perform functional annotations and interpretations, including gene ontology (GO) and kyoto encyclopedia of genes and genomes (KEGG) analysis. Fisher’s exact test was used to select the significant terms and pathways. P < 0.05 and FDR < 0.25 were considered as significantly enriched. Results of these functional annotations were visualized by circus plot, chord plot or circle plot, respectively.

### Statistical Analysis

Data statistical analysis and visualization were performed by R (version 4.1.2, based on different packages mentioned above) and Graphpad Prism (version 8.3.0). The association between continuous variables were assessed by Spearman’s correlation coefficient. When three or more groups to compare, flexibly chose one-way ANOVA analysis, and student t test was applied for statistical analysis between two groups. P < 0.05 was considered as significantly statistical difference.

## Results

### Construction of Weighted Gene Co-Expression Network

**Figure 2:**
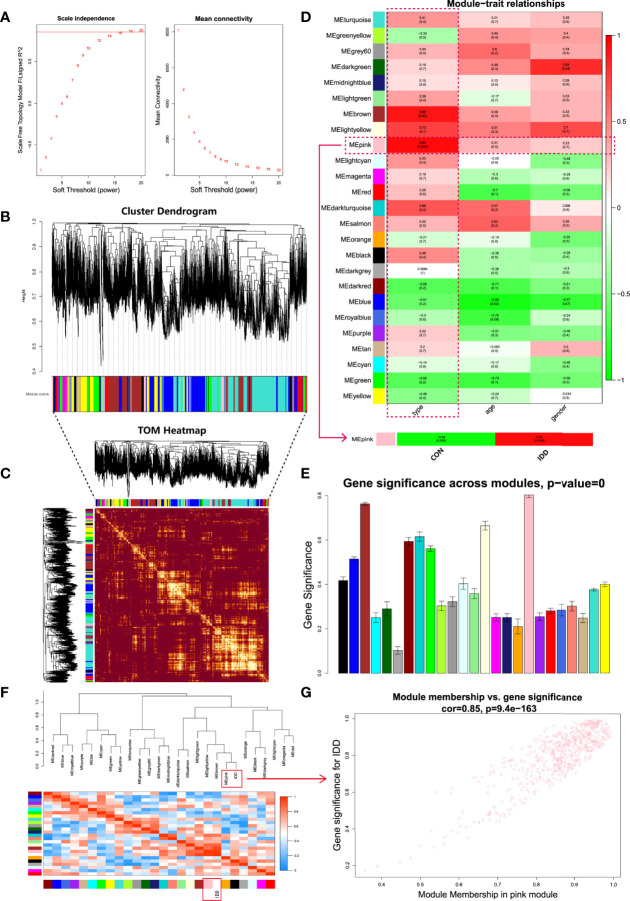
Comprehensive weighted gene co-expression network analysis. **(A)**, Selection of soft threshold power value. Left panel displayed scale-free model fit index of different power values; right panel indicated the mean connectivity of these values. **(B)**, Clustering dendrogram of all genes based on dissimilarity algorithm and assignment modules. **(C)**, Topological overlap heatmap of the weighted co-expression network. Each row and column represented genes, different colors in x and y axis represented different clustered modules. Light area indicated high topological overlap within network. **(D)**, Module-trait correlation heatmap between clinical traits and modules. **(E)**, Histogram of gene significance across modules showed pink module was the most significant module in degenerative CEP group. **(F)**, Cluster dendrogram and heatmap of adjacency eigenvalue in the network. **(G)**, Correlation scatter plot between gene significance and module membership in pink module.

Heterogeneity of each sample was detected by hierarchical clustering analysis to eliminate outliers from study, and all samples were included for subsequent research. Totally all 18800 genes and 6 samples in gene expression matrix were applied for WGCNA analysis, avoid using the top 5000 genes to get an optimal and comprehensive clustering analysis. As shown in **Figure 2A**, soft threshold power β was determined as 18 when R^2^ reached 0.90 and mean connectivity approximately approached 0. Consequently, β was determined as 18 to construct the following weighted co-expression network.

Based on the weighted network and gene mutual co-expression situation, we conducted hierarchical clustering tree analysis, to cluster genes which could interact with each other, modules were generated with the most similar expression situation. Altogether, 25 modules were obtained based on their expression profile (**Figure 2B**). Dendrogram branches indicated that genes in each module were highly heterogenous and TOM heatmap (**Figure 2C**) validated that each co-expression module could independently distinguish each other within network. Next, the characteristics between these generated modules and clinical features were further analyzed in each subgroup.

### Key Module Identification of CEP in DDD

After construction of WGCNA network, we then evaluated relationships between different modules and clinical traits (disease state, age and gender). Heatmap revealed the correlations between different modules and clinical features, as shown in **Figure 2D**, results visualized that gene modules were independent from age and gender (P all > 0.01), but were highly correlated with the disease state: Pink (P = 0.005) and brown (P = 0.02) modules had the most correlations with disease state. Then we further extracted the pink module and analyzed it with the disease state (normal and DDD group). Ulteriorly, in the column of DDD group, pink module had the highest correlation (r = 0.94) and the most significant difference (P = 0.005) with degenerative CEP. Thereinto, genes in pink module were considered as the highly active genes to regulate the development of degenerative CEP in DDD. Gene significance histogram (**Figure 2E**) of each module proved the reliability of our results that pink module was the most correlated one. We further calculated module eigenvalue adjacency of both modules and clinical traits, similar eigenvalues were clustered together. As shown in eigenvalues heatmap of **Figure 2F**, results visualized that pink module clustered with degenerative CEP together, implying that genes in pink module could interact with degenerative CEP whether by activation or suppression. These above results all prompted us to select pink module as the interested module and applied for further analysis.

**Figure 3:**
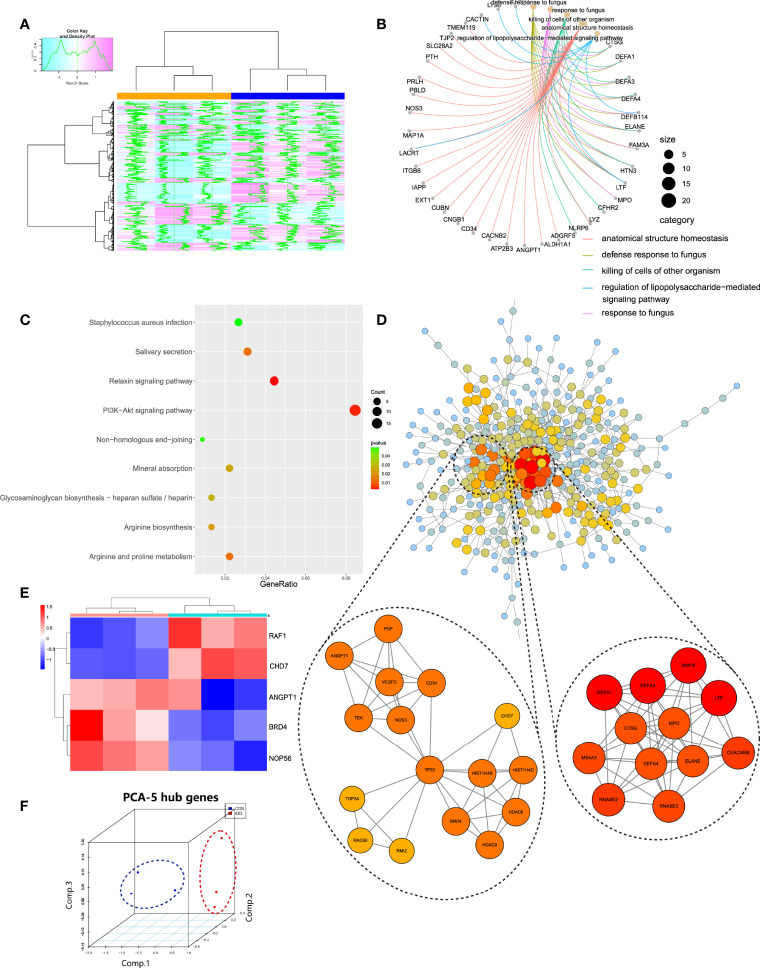
**(A)**, Pink module genes expression heatmap among normal and DDD groups. **(B, C)**, Functional and pathways enrichment analysis of pink module. **(D)**, Construction of PPI network based on genes in pink module, and the top 2 related sub-networks by MCODE algorithm. **(E)**, Hub genes expression heatmap identified in pink module. **(F)**, 3D scatter plot after reducing dimension of hub genes by PCA method.

Based on pink module, this study performed scatter plot of MM-GS (module membership vs. gene significance) to further observe the overall variation trend of genes in degenerative CEP (**Figure 2G**), results displayed that genes in pink module had a positive correlation and low P value (Cor = 0.85, P = 9.4e-163) with degenerative CEP, indicating a good linear relationship. Then we visualized these genes in heatmap to further figure out the separating capacity between normal and degenerative CEP group, results showed a pretty distinguishment (**Figure 3A**).

### Functional and Pathway Enrichment Analysis in WGCNA

Pink module totally contained 579 genes, then GO and KEGG analysis was conducted to get a comprehensive understanding of the biological functions and aberrant signaling pathways in the development of degenerative CEP based on these genes. As shown in **Figure 3B**, results suggested that these active genes were highly involved in several dysfunctions of cartilage/bone development, like killing of cells of the other organism, innate immune response in mucosa, anatomical structure homeostasis etc., and some functions to induce IVD homeostasis like tissue homeostasis; KEGG results (**Figure 3C**) also analyzed several abnormal signaling pathways to induce IVD deterioration, such as relaxin signaling pathway and Mineral absorption procedure etc., and other pathways that promoted IVD development, including arginine and proline metabolism and Glycosaminoglycan biosynthesis procedure, and PI3K-Akt signaling pathway. These functions and pathways both behave different roles in regulating homeostasis and instability of CEP in DDD. The detailed information of genes in pink module as well as enrichment functions were provided in **Supplementary Table 1**.

### Selection of Hub Genes by PPI Construction

Genes in pink module were analyzed in STRING database to generate the PPI network, which was then uploaded into Cytoscape to further construct connective sub-network as well as identifying hub genes. As shown in **Figure 3D**, totally the network containing 406 nodes and 818 edges were generated according to PPI construction. Correlated sub modules were then analyzed by ‘MCODE’ plug-in in Cytoscape, 2 main sub modules were acquired: module 1 contained 12 nodes and 53 edges and module 2 had 16 nodes and 43 edges, where genes in these modules had the highest correlations, and could jointly regulate the occurrence of degenerative CEP. Their functions were also evaluated by GO, KEGG, as listed in **Table 1**: module 1 were mainly involved in functions of killing of cells of other organisms and ECM disassembly; signaling pathways were focused on transcriptional mis-regulation in cancer and NOD-like receptor signaling pathway; and module 2 behaved roles in regulating angiogenesis and tissue remodeling such as sprouting angiogenesis, regulation of vasculature development, and some activated signaling pathways focused on classical PI3K-Akt and MAPK signaling pathways etc. Then, hub genes in this network were filtered by ‘cytohubba’ algorithm, 5 genes including BRD4, RAF1, ANGPT1, CHD7 and NOP56 were both existed in most of 11 different methods after calculating, which were finally identified as hub genes. The gene expression levels of these hub genes were further tested and displayed in heatmap (**Figure 3E**), results visualized a pretty distinguishment ability of these hub genes, indicating they may be pivotal in the pathogenesis of degenerative CEP in DDD. Then after dimensional reduction of these four hub genes by PCA method, 3 components were generated: PC1, PC2 and PC3, three dimensional spatial coordinate system illustrated that different types of samples were clustered together in spatial distribution (**Figure 3F**).

**Table 1:** Functional and pathway (GO, KEGG) enrichment analysis of MCODE identified sub-networks.

Module	Term	Description	P value	Count
1	GO:0002251	organ or tissue specific immune response	6.88E-14	6
	GO:0031640	killing of cells of other organisms	1.66E-12	6
	GO:0001906	cell killing	8.32E-10	6
	GO:0022617	extracellular matrix disassembly	7.82E-06	3
	GO:0002251	organ or tissue specific immune response	6.88E-14	6
	hsa05202	Transcriptional misregulation in cancer	1.44E-07	5
	hsa04621	NOD-like receptor signaling pathway	3.717E-04	3
2	GO:0010634	positive regulation of epithelial cell migration	1.79E-09	6
	GO:0045766	positive regulation of angiogenesis	1.49E-07	5
	GO:0002040	sprouting angiogenesis	1.66E-07	5
	GO:1901342	regulation of vasculature development	3.76E-06	5
	GO:0048771	tissue remodeling	0.000270841	3
	hsa04926	Relaxin signaling pathway	0.014817629	3
	hsa04151	PI3K-Akt signaling pathway	4.79E-06	6
	hsa04010	MAPK signaling pathway	3.81E-05	5

### Immune Infiltration Landscape in CEP Tissue

**Figure 4:**
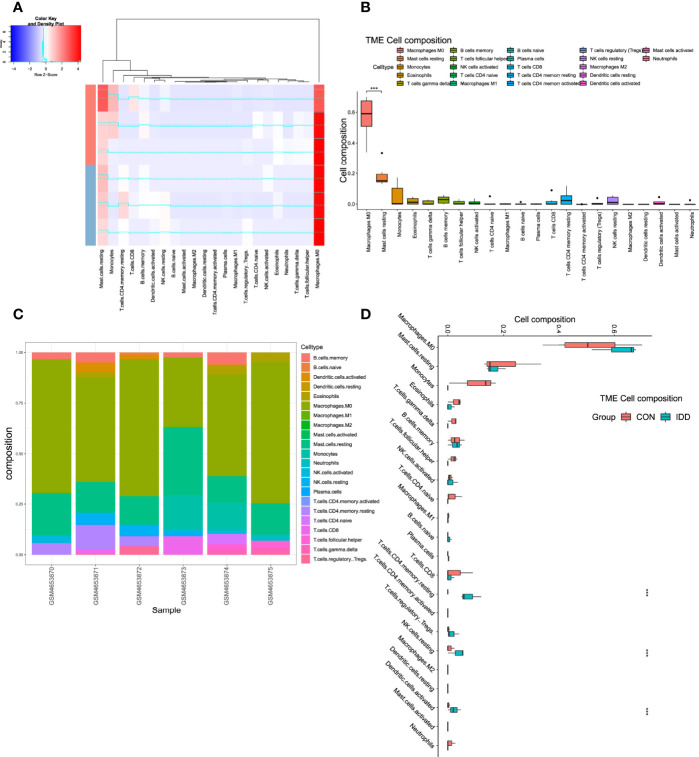
Analysis immune landscape associated with DDD in CEP tissue. **(A)**, Heatmap visualizing the distribution of 22 types of immune cells in CEP tissue from normal and DDD patients. **(B)**, Box plot showing the whole composition of immune cells in these tissues. **(C)**, Pile-up histogram displaying proportion of immune cells in each sample. **(D)**, Differentially analysis of immune infiltration levels between normal and DDD patients.

Former enrichment analysis suggested that some immune-related functions and signaling pathways were aberrantly activated in degenerative CEP tissues. This study further discovered the different situations of immune landscape based on bulk-RNA seq data between CON and DDD patients. ‘CIBERSORT’ method was performed *via* deconvolution algorithm, which was conducted to calculate the abundance of immune cell members from mixed cell populations. **Figure 4A** displayed the correlated clustered information and distribution of 22 types of immune cells in CEP tissue; detailed proportion situations of immune cells in each sample were clearly shown in **Figures 4B, C**, results visualized that the M0 macrophage cells and mast cells occupied for the main components in both normal and degenerative CEP tissues, while M1 and M2 macrophages seldomly expressed in both tissues. Furthermore, the immune landscape elucidated that CD4^+^ memory T cells, NK cells and dendritic cells were highly activated in degenerative CEP tissue (P < 0.001), while M0 macrophages displayed no significant difference (P > 0.05) (**Figure 4D**).

### Single-Cell Profiling Atlas Revealed Highly Cellular Heterogeneity in CEP Tissue

**Figure 5:**
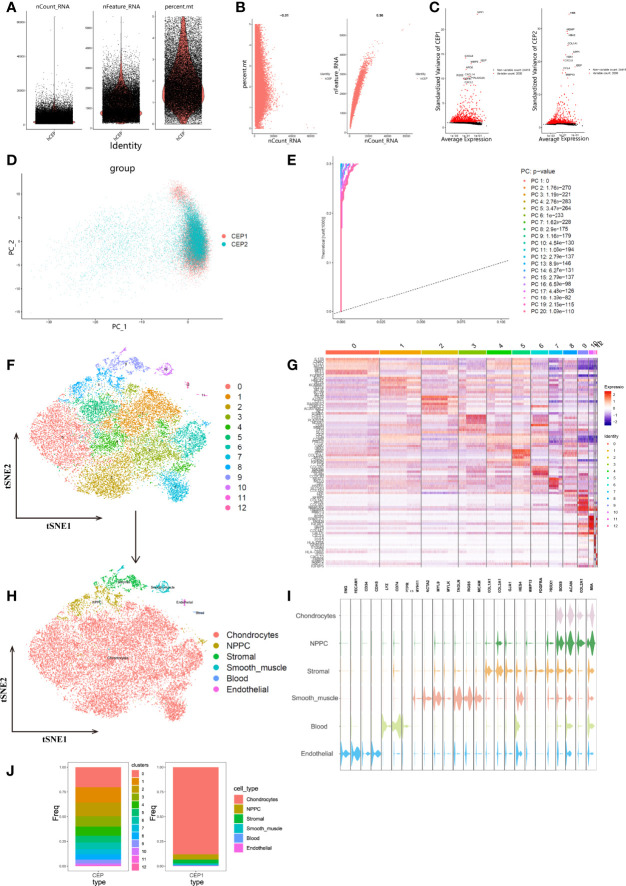
**(A)**, After standard quality control of all cells from CEP tissue of 2 patients, 26209 cells were included in the analysis. **(B)**, The numbers of detected genes were significantly related to the sequencing depth, with a high Pearson’s correlation coefficient 0.96; the numbers of detected mitochondria were the same among different sequencing depth. **(C)**, Variance diagram showed 26418 genes throughout all cells from CEP tissue. Red dots represented highly variable genes and black dots represented non-variable genes. The top 10 most variable genes in each tissue were marked in scatter plot. **(D)**, PCA scatter plot displayed dots distribution after integration analysis, which did not show clear separations of cells. **(E)**, PCA algorithm identified the 20 PCs with an estimated P Value < 0.05. **(F)**, tSNE dimensional reduction method was applied with the top 30 PCs, and 13 cell clusters were classified. **(G)**, Differentially analysis revealed 4295 marker genes. The top 5 genes for each cluster were displayed in heatmap. **(H)**, tSNE plot after cell type annotation for each cluster. **(I)**, Violin plot showed the mean expression of selected marker genes used for annotation in each cell type. **(J)**, Proportion of each cluster and cell type in CEP tissue.

To determine the single-cell level transcriptomic landscape of IVD composition, we employed scRNA-seq data from CEP tissues. Following the rigorous quality control criteria, low quality cells with high mitochondria UMI rate (> 5%) were excluded, and totally 26209 CEP cells were selected for subsequent analysis (**Figure 5A**). Mitochondria UMI rate of each cell were lower than 5%, and the number of genes detected were significantly highly related to the sequencing depth (**Figure 5B**). A total of 26418 genes were analyzed and the variance analysis revealed the top 2000 highly variable features in **Figure 5C**. PCA method was then conducted to identify available dimensions and filter correlated features, dot plots and heatmap displayed the top 20 significantly correlated genes (**Supplementary Figure 1**). After integration of data by RPCA algorithm, PCA scatter plot showed a high overlap among cells in human CEP tissues (**Figure 5D**), demonstrating a good integration result. We then calculated 50 principal components (PCs) of the top 2000 genes based on the estimated P values, and this study finally chose the top 30 PCs with highest P values for subsequent analysis, **Figure 5E** displayed the top 20 PCs.

Unsupervised analysis was then conducted for cell population clustering through tSNE algorithm, totally 13 cell clusters were generated in CEP tissue, and different cell populations showed high heterogeneity with each other (**Figures 5F, G**). The detailed cell types were identified according to the expression patterns of marker genes, which were referenced by singleR (31), CellMarker (31), and classification results of Gan et al. (32). Altogether 6 categories were annotated, including mesenchymal chondrocytes, nucleus pulposus mesenchymal stem/progenitor cell (NPMSC or NPPC), stromal, smooth muscle/pericyte, blood and endothelial cells. The expression patterns of these marker genes were tested and annotated as following (**Figures 5H, I**): endothelial cells were consist of cluster 12, with specific marker genes ENG, PECAM1, CD34 and CDH5; blood cells were consist of cluster 11 with marker genes LYZ, CD74 and PTPRC; the marker genes of cluster 10 included MYH11, ACTA2, MYL9, MYLK, TAGLN, RGS5, MCAM, which identified smooth muscle/pericyte cells; cluster 9 were annotated as stromal cells, based on marker genes of COL1A1, COL3A1, GJA1, HES4 and MMP13; cluster 8 were annotated as NPMSC for marker genes PDGFRA and PRRX1; the rest of clusters 0-7 were regarded as chondrocytes, for highly expressed marker genes of SOX9, ACAN, COL2A1, MIA, etc. The proportion of cell populations before and after annotations were compared in **Figure 5J**.

**Figure 6:**
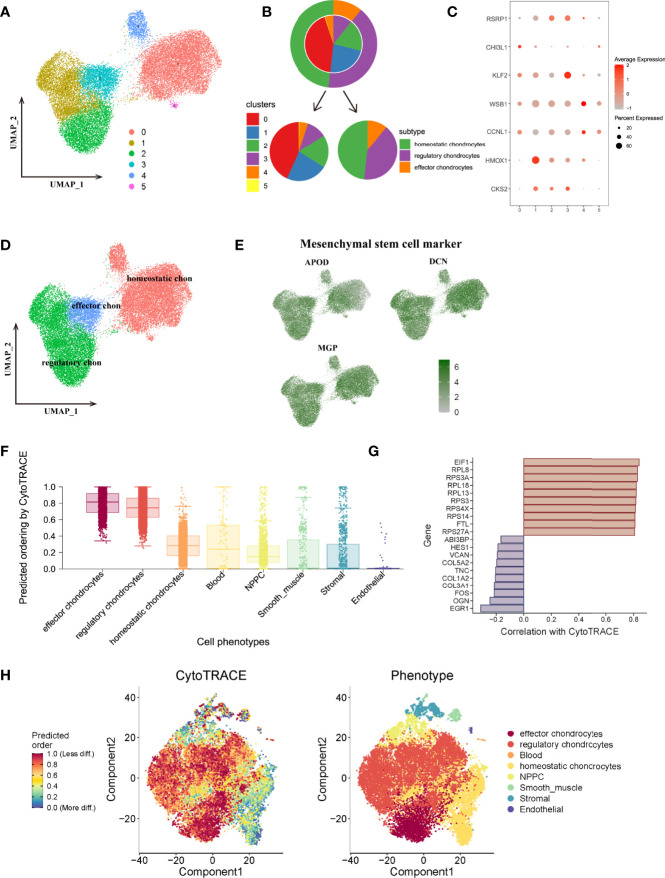
**(A)**, UMAP plot of chondrocytes subtypes, which were extracted from the whole cells and applied for re-UMAP analysis. **(B)**, Sector proportion diagram of chondrocytes before and after cell type annotations. **(C)**, Dot plot displayed the mean expression of selected marker genes among different clusters. **(D)**, UMAP plot after cell type annotation for each cluster. **(E)**, the average expression of mesenchymal stem cell markers for chondrocytes on the tSNE map. **(F)**, Plot of the cytoTRACE pseudotime order for the CEP subpopulations. The value of each cell type in cytoTRACE represented the predicted order. **(G)**, The top 10 correlated genes in the overall cell type differentiation. **(H)**, Overall tSNE plot for each cell based on cytoTRACE algorithm, blue dots represented low differentiated state while red dots represented high differentiated state for cells.

To better understand and subdivide the detailed cell types of mesenchymal chondrocytes, this study further extracted chondrocytes and performed re-analysis with tSNE and UMAP, and chondrocytes were re-clustered into 6 clusters (**Figure 6A**). We analyzed the marker genes functions and refereed to the clustering information of Gan et al, finally clustered chondrocytes into 3 subtypes, the proportion of different subtypes and the expression situation of marker genes in each cluster and were shown in **Figures 6B, C**, including homeostatic chondrocytes: clusters 0,4,5 (marker genes of CCNL1, WSB1), regulatory chondrocytes: clusters 1,2 (marker genes of CKS2, HMOX1) and effector chondrocytes: cluster 3 (marker genes of KLF2, CHI3L1, RSRP1), as shown in **Figure 6D**. Besides, all chondrocytes highly expressed mesenchymal stem cell marker genes APOD, DCN, MGP, thus, these chondrocytes were regarded as subgroups with potential differentiated functions (**Figure 6E**).

### Functional Enrichment of Different Cell Populations

**Figure 7:**
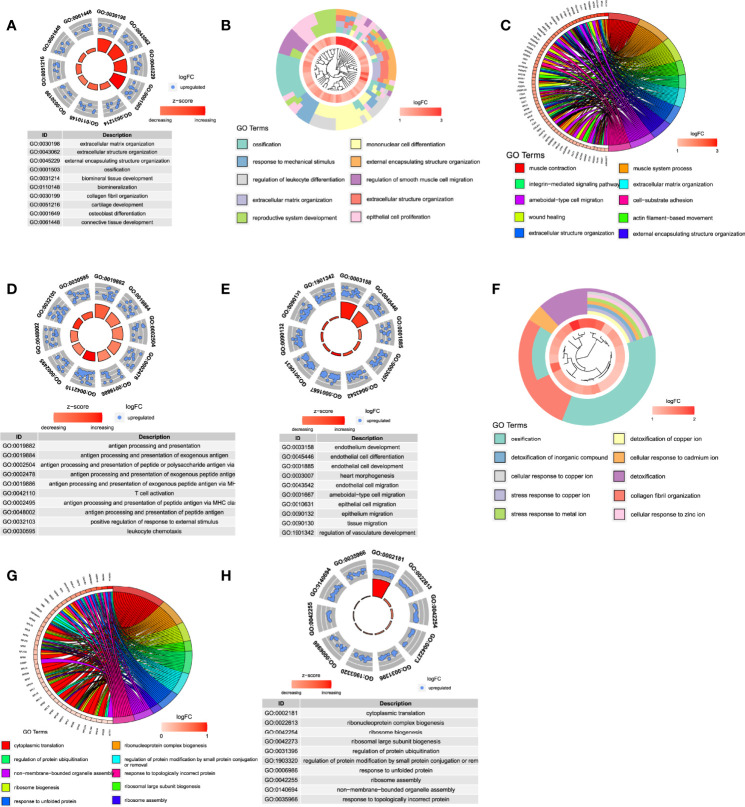
Functional enrichment analysis of different cell populations. **(A–H)**, The top 10 function enrichment analysis for NPMSC, stromal cell, smooth muscle cell, blood cell, endothelial cell, homeostatic chondrocytes, regulatory chondrocytes, and effector chondrocytes, respectively.

To obtain a comprehensive understanding of the biological functions and aberrant signaling pathways of different cell populations in CEP tissue, ‘FindMarkers’ function was performed to obtain DEGs among different cell types (see in **Supplementary Table 2**). GO and KEGG analysis were then conducted based on DEGs of different cell types, results (KEGG illustration see in **Supplementary Figure 2**) indicated that NPMSC cells (**Figure 7A**) mainly activated in ECM organization, ossification, biomineral tissue development, cartilage development, and ECM-receptor interaction, which were consistent with the characteristic that behaved roles as mesenchymal stem cells, indicating NPMSC cells may contribute to promoting and maintaining bone/cartilage formation; Stromal cells (**Figure 7B**) chiefly contribute to ossification, response to mechanical stimulus, smooth muscle cell migration, epithelial cell proliferation etc., suggesting stromal cells helped maintain spinal stabilization; meanwhile, abundant immune cell-related functions were activated, like T cell activation, T and B cells differentiation, proliferation, macrophage migration and chemotaxis etc., which could also be served as environment place for these immune cells growth, to interact with blood cells and eventually promote immune infiltration procedure.

Blood-related cells existed in CEP tissue included smooth muscle cells, blood cells and endothelial cells, among which, smooth muscle cells (**Figure 7C**) behaved roles like muscle contraction, regulation of angiogenesis, regulation of vasculature development through PI3K-Akt signaling pathway, which provided a hard evidence that small amount of blood vessels existed in CEP, nutrition could be transported and diffused into NP and AF tissues through blood vessels in CEP; As for blood cells (**Figure 7D**), they were focused on antigen processing and presentation, and lots of immune cell-related functions, like T, B, and macrophage cells activation, cytokine-mediated signaling pathway and NF-κB signaling pathway etc. Endothelial cells (**Figure 7E**) also behaved several functions like smooth muscle and blood cells, such as vasculo-genesis, epithelial cell proliferation and regulation of angiogenesis, some were activated through PI3K-Akt signaling pathway, these above functions suggested that blood-related cells could interact with cytokines to further regulate the degeneration of CEP, AF and NP through blood vessels infiltration, thus, IVD is prone to degeneration is not difficult to understand.

In terms of mesenchymal chondrocytes, they were further classified into 3 parts: homeostatic, effector and regulatory chondrocytes. Homeostatic chondrocytes (**Figure 7F**) mainly involved in functions of ossification, collagen fibril organization, and collagen binding etc.; while regulatory chondrocytes (**Figure 7G**) were enriched for several signaling regulation and stimulus reactions like response to temperature stimulus and regulation of apoptotic signaling pathway etc.; as for effector chondrocytes (**Figure 7H**), results indicated a significant enrichment of metabolic processes and positive regulation of intrinsic apoptotic signaling pathway etc., which were thus considered as effector cells with high metabolic rates and protective/restore functions. The detailed results of all functional and pathway information about each cell population were shown in **Supplementary Tables 3**, **4**.

### Cell Fate and Pseudotime Analysis Indicated Mesenchymal Chondrocytes Were Active Subgroups in Regulating Cell Differentiation

To further obtain a comprehensive view of differentiation states and potentials of each cell population, we then performed cell fate analysis based on all cell populations in CEP tissue. Cellular trajectory reconstruction analysis using gene counts and expression (cytoTRACE) could reveal the direction of differentiation and predict cell lineage trajectories (27). As shown in **Figure 6F**, results calculated and displayed the order of the differentiation states as effector chondrocytes, regulatory chondrocytes, homeostatic chondrocytes, blood, NPMSC, smooth muscle, stromal and endothelial cells. The detailed differentiation states of each cell were visualized in cytoTRACE tSNE plot (**Figure 6H**), among which the top 10 related genes mediating cell differentiation were EIF1, RPL7, RPS3A, RPL18, RPL13, RPS3, PRS4X, PRS14, FTL, RPS27A, as shown in **Figure 6G**. Results also demonstrated that effector, regulatory and homeostatic chondrocytes had the highest cytoTRACE scores, indicating a better differentiation potential than other cell types. These results were consistent with our previous findings that effector and regulatory chondrocytes behaved essential roles in regulating aberrant signaling pathways and may act as active subgroups mediating different cell fates.

**Figure 8:**
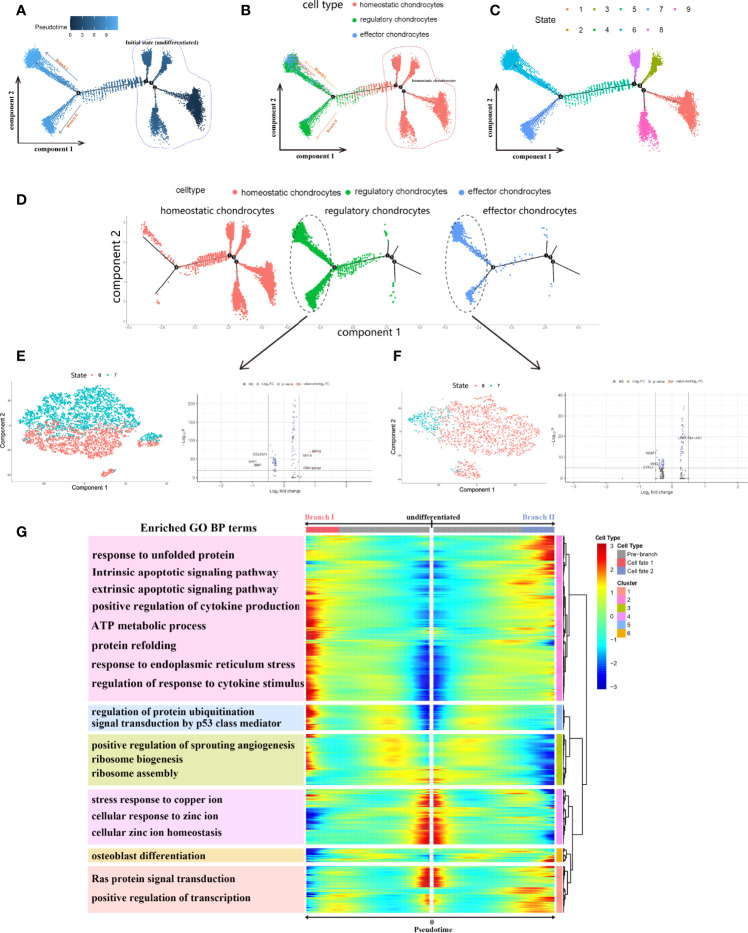
Monocle pesudotime trajectories analysis revealed the chondrocytes cell lineage progression. **(A)**, Trajectory differentiation diagram based on cell differentiated state, dark dots represented the initial state (marked by circle) and light dots represented different 2 cell fates (branch I and II). **(B, C)**, Trajectory differentiation diagram colored by cell types and differentiated states. Cells within initial state were homeostatic chondrocytes. **(D)**, the detailed distribution of different chondrocytes in trajectory plot during differentiation. **(E, F)**, PCA dimension reduction of regulatory and effector chondrocytes based on state 6 and 7, respectively, and differential analysis of genes. **(G)**, Branch trajectory heatmap of the DEGs revealed the gene alterations under different cell differentiation states. The main functions of genes in each cluster were also displayed.

In order to further investigate the detailed cell trajectory of chondrocytes, we conducted pseudotime analysis. All chondrocytes were projected into trajectory and generated 1 root area and 2 branches, termed as branch I and II (**Figures 8A-C**). The results of pseudotime demonstrated that homeostatic chondrocytes served as progenitor cells, were almost located together at the root area, while effector and regulatory chondrocytes located at different branches I and II from node 4. Different states indicated different cell fate, suggesting that cells in different branches may behave different roles in the development of CEP (**Figure 8D**). We wondered the detailed gene expression alterations of these chondrocytes during differentiation, then BEAM analysis was performed for branch fate-determined gene analysis according to node 4. As shown in **Figure 8G**, branch heatmap visualized significant gene expressed changes in different differentiation directions, cell fate I (branch I) represented the smaller state ID (state 6) and cell fate II (branch II) represented bigger ID (state 7), DEGs were clustered into 6 categories and the enriched functions were illustrated in branch heatmap, where genes in clusters 2,3,5 were upregulated in cell fate I while clusters 1,6 were activated in cell fate II through the differentiation processes, genes alterations in cluster 4 remained consistent in the pseudotime process. GO BP terms indicated that DEGs of clusters 1,6 were activated in functions like osteoblast differentiation, Ras protein signaling transduction and positive regulation of transcription, which contribute to bone/cartilage growth and restore the homeostasis of CEP in IVD; while DEGs in clusters 2,3,5 were chiefly involved in response to unfolded protein, intrinsic and extrinsic apoptotic signaling pathway, protein refolding, positive regulation of sprouting angiogenesis etc., which may induce the degeneration of CEP. Monocle dimension reduction analysis further validated our results, that both regulatory (**Figure 8E**) and effector chondrocytes (**Figure 8F**) could be significantly classified into two types according to cell state (6 and 7). After differential genes analysis, regulatory chondrocytes had several different DEGs mediating different cell fate, including COL10A1, SPP1, IBSP, MT1G, MT1X, OGN etc.; and effector chondrocytes had DEGs such as NEAT1, MEG3, CYTL1 which may also behave different functions in inducing effector chondrocytes differentiation. The two branches balanced with each other and once the state was disrupted, different cellular functions as well as cell fates may occur.

### Cell-Cell Communication Analysis Revealed Cascade Reactions From Different Populations in the Development of CEP

**Figure 9:**
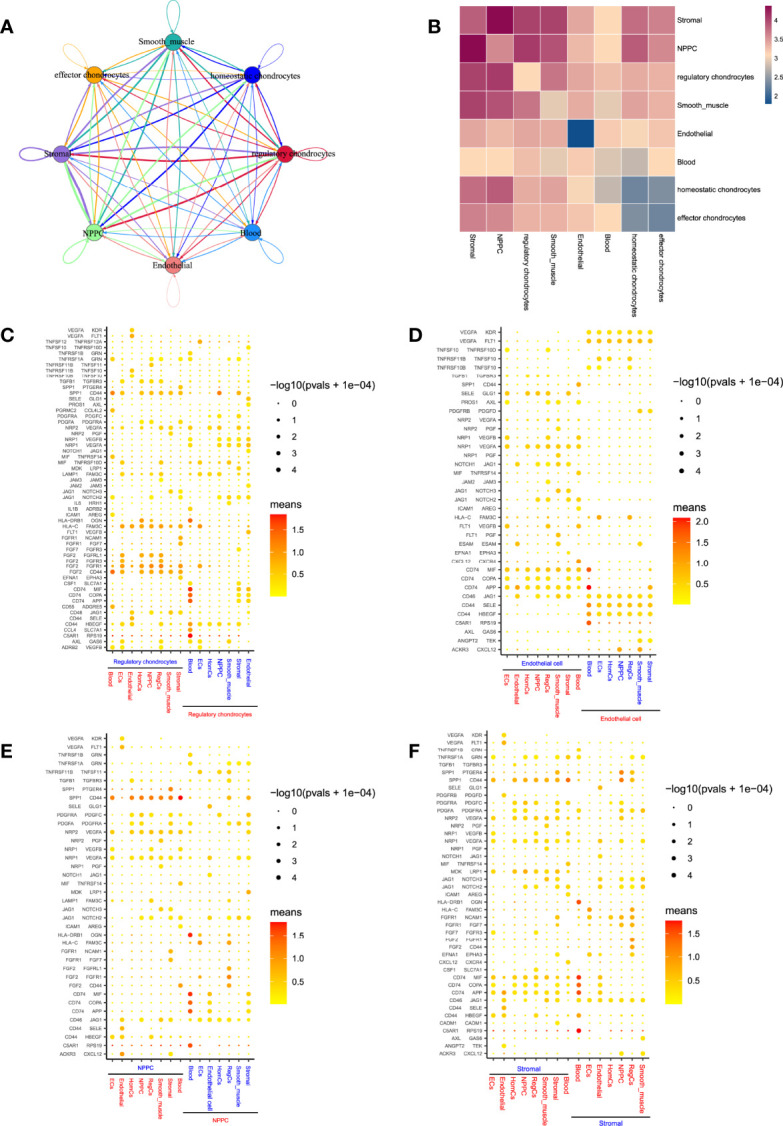
Predicted cell populations regulatory network in CEP. **(A)**, Overall view of the cellular inter-regulatory network. Dot size indicated the relative quantity of each cluster; line thickness represented the relative quantity of significant ligand-receptor pairs. **(B)**, Correlation heatmap revealed the number of potential ligand-receptor pairs between cell groups. **(C–F)**, Bubble plots showing each statistically significant ligand-receptor pair in regulatory chondrocytes, endothelial cells, NPMSC and stromal cells, respectively.

To obtain insights into the critical factors involved in the CEP cell niche of human IVD, this study performed CellPhoneDB analysis to discover the possible means of different cell communications in CEP tissue. Relatively active bidirectional interactions among those cell populations displayed highly regulated cellular communications (**Figure 9A**). Communication correlation heatmap (**Figure 9B**) demonstrated that regulatory chondrocytes showed the most interactions with other cell types, indicating an active subtype from chondrocytes, which was consistent with our findings in cytoTRACE analysis; besides, NPMSC and stromal cells also displayed high connections with other populations. Thus, regulatory chondrocytes, NPMSC and stromal cells were determined as niche components in the CEP tissue, which behaved essential roles to regulate the differentiation processes through cascade signaling interactions. Based on above findings, this study hypothesized that NPMSC, stromal and chondrocytes could interact and regulate with each other, both may behave promotion or degeneration process in bone/cartilage formation and blood vessels infiltration through different processes. The detailed interactions situations between each cell type were illustrated in **Supplementary Figure 3**.

In regulatory chondrocytes-related cell communications (**Figure 9C**), SPP1/CD44, CD44/HBEGF and HLA-C/FAM3C were widely existed in the interactions between regulatory chondrocytes and other cells; besides, regulatory chondrocytes also produced VEGFA, which bind to VEGF receptors (KDR, FLT1, NRP1, and NRP2) in endothelial cells or in regulatory chondrocytes through paracrine or autocrine. In more cases, regulatory chondrocytes acted as receptors to receive angiogenesis regulation from other cell types. The endothelial cells-related communications (**Figure 9D**) also displayed that endothelial cell were involved in VEGF signaling, and served as receptors to regulate angiogenesis through VEGFA/KDR or VEGFA/FLT1 signaling pathways, which implied that the interactions between these cell types and endothelial cells were potentially proangiogenic. Notably, healthy human IVD was largely avascular organ, angiogenesis occurred in NP tissue during degeneration (33), this study also provided the evidence of the possibility of blood vessels infiltration in CEP tissue.

In NPMSC and stromal cells (**Figures 9E, F**), It has also indicated that they produced SPP1, MIF and their receptors, SPP1/CD44 signaling pathway was widely activated in interactions with chondrocytes, SPP1 served as an osteogenesis-related factor, was significantly involved in the interactions among stromal and NPMSC cells in CEP tissue, which contributed to the osteoblast differentiation (34). However, CD74/MIF signaling was regulated by stromal cells and acted on chondrocytes, MIF was reported to promote osteoclast differentiation (35). Besides, NPMSC and stromal cells both expressed growth factor PDGF, showing pro-proliferation effects on homeostatic chondrocytes, NPMSC and stromal cells themselves, while almost no effects on regulatory and effector chondrocytes, indicating the different cell states between homeostatic and effector/regulatory chondrocytes.

Overall, our results depicted a network among different cell populations, which may interact with each other in the balance of inflammation, cell proliferation and angiogenesis within CEP tissue. Additionally, these results were in line with our above hypothesis that NPMSC, stromal and chondrocytes may behave essential roles in IVD homeostasis or degenerative processes. Even if in the normal IVD tissue, degenerative changes may occur during different cellular regulatory processes, no wonder most elderly patients eventually experienced DDD. However, the exact mechanisms need to be conducted by further experiments.

## Discussion

DDD served as a severe threat to human health and social economics, has consistently affected individual’s back pain (36). Inadequate knowledge of the physiology and pathology of DDD all prompted researchers to seek more innovative approaches and effective diagnostic targets in the treatment of DDD, among which genetic alterations were the most essential part (10, 11). Relevant single-cell studies of NP and AF tissues provided promising transcriptomic landscape of different cell niches in the regulation of NP or AF tissue (15, 16, 32, 37). While CEP, although regarded as the unimpressive structure in IVD, also behaved pivotal roles in regulating the homeostasis of IVD and had tight connections with DDD. However, related mechanisms between different cell populations in CEP were not well studied, relevant researches had focused more on the CEP as whole (38), with few knowledges about communication network within different cell types. Therefore, due to cellular heterogeneity and complex microenvironments within IVD, it’s an urgent need to perform novel single-cell analysis, to realize specific biomarkers and interactions among different cell types in the homeostasis of CEP.

WGCNA served as a powerful holistic research tool, has been widely analyzed for the relationships between clinical features and gene expression profiles (39), to the knowledge, the application of WGCNA in the identification of degenerative CEP had not been well studied, also, few researches had focused study on CEP tissue. In our study, we conducted a first conjoint analysis with WGCNA and single-cell analysis to explore the underlying mechanisms of degenerative CEP, and also investigated cellular heterogeneity among CEP tissue. Here, we explored and provided a group of gene signatures which behaved essential roles in the development of degenerative CEP for researchers to focus on, then we constructed a transcriptomic landscape of CEP components, we found that regulatory and effector chondrocytes both had dual roles in maintaining the homeostasis of CEP. Besides, this study had fully analyzed the underlying interactions of each cell population, and their different destination within the differentiation.

In WGCNA analysis, most researches chose the top 5000 genes based on the median absolute deviation (MAD) for subsequent network construction, due to the computing power of computer. While in this study, we included all genes for WGCNA analysis, to avoid missing any gene information. Totally 25 distinct co-expression modules were generated based on weighted gene network, among which genes in pink module had the lowest P values as well as the highest correlation coefficients, which was finally determined as the most correlated module in the progression of degenerative CEP. The module-trait relationship and module-eigenvalue adjacency heatmap confirmed our results. Additionally, hierarchical clustering analysis also illustrated that genes in pink module could distinguish degenerative CEP from normal group significantly.

Functional and pathway enrichment analysis about pink module helped us understand the underlying mechanisms for degenerative CEP. Functional annotation indicated that these genes were mainly enriched in killing of cells of the other organism, innate immune response in mucosa, anatomical structure homeostasis, which may explain why CEP was prone to degeneration. Then with the aim of screening hub genes in pink module, the interactive network of 406 mutual genes were constructed by PPI based on STRING database, after ‘cytohubba’ algorithm, 5 genes (BRD4, RAF1, ANGPT1, CHD7 and NOP56) were determined as hub genes responsible for the development of degenerative CEP. PCA 3D scatter plot and heatmap further validated their distinguishment ability.

Recent exploited novel integration algorithm in preprocessing single-cell data, including iNMF algorithm developed by Gao et al. (26), and DAVAE and VIPCCA algorithms developed by Hu et al. (24, 25) all demonstrated promising effects in single-cell data process, such as data alignment and integration by multiple samples and different datasets, which could processed more cells at once with less consumption of computer resource than Seurat, like runtime of calculating or RAM usage of computer. Thus, these methods were strongly recommended when processing multiple modalities or massive different datasets. In terms of constructed transcriptomic atlas of CEP tissue, this study used RPCA integration algorithm (based on conventional Seurat v4) to eliminate batch effects for small amount of cells, about 27000. Totally 13 cell clusters were identified in CEP, 1 cluster were NPMSC cells, 1 cluster were stromal cells, 1 cluster were smooth muscle/pericyte cells, 1 cluster were endothelial cells, 1 cluster were blood cells and the rest of 8 clusters were identified as mesenchymal chondrocytes, which were further classified into 3 sub chondrocytes: regulatory, effector and homeostatic mesenchymal chondrocytes based on their highly activated genes functions.

During cartilage development, chondrocytes undergo terminal differentiation during hypertrophic, it is widely believed that hypertrophy-like changes in chondrocytes played roles in osteoarthritis (40). Besides, it had been reported that hypertrophic differentiation occurred in NP tissue during the progression of DDD (41). Thus, this study performed cell fate analysis to further determine the differentiation states of chondrocytes in CEP tissue. CytoTRACE analysis revealed that regulatory chondrocytes had the highest scores, indicating a better differentiation potential than other cell types, among the top 10 related genes, ribosomal proteins accounted for the majority in the development of cell differentiation. Studies reported that ribosomal proteins had extra-ribosomal functions like regulating cell development, differentiation, and DNA repair, and they were also associated with cancer, aging and age-related degenerative diseases (42, 43), which demonstrated the active states of regulatory chondrocytes. More detailed functions and mechanisms of these ribosomal proteins in regulating chondrocytes were worth analyzing in the development of CEP. During cell differentiation trajectory, chondrocytes began with homeostatic and ended with two different cell fates with regulatory and effector chondrocytes based on node 4. We observed that genes in two different branches behaved opposite roles in regulating bone/cartilage development: in branch I direction, genes were mainly associated with the degenerative processes of CEP, while in branch II direction, genes were mostly enriched in regulating osteoblast differentiation and homeostasis of CEP. Besides, the PCA results based on two branches also demonstrated our conclusion, chondrocytes in state 6 and 7 were significantly different, among which regulatory chondrocytes had DEGs like SPP1, IBSP, MT1G, MT1X, OGN etc., and effector chondrocytes had DEGs such as NEAT1, MEG3, CYTL1, which were needed for further researches. These findings indicated that both regulatory and effector chondrocytes had potential opposite effects in differentiation trajectory, through regulating bone/cartilage development or inducing degeneration processes. The opposite functions of these two cell mesenchymal chondrocytes balanced with each other and once the state was disrupted, different cellular functions as well as cell fates may occur, no wonder most elderly patients suffered DDD eventually. More experiments need to be conducted to further validate our results.

As the largest avascular organ in body, the nutrient supply approaches of IVD are special, the nutrition and metabolic products are carried out through blood vessels outside the disc, nutrients supplied by capillaries and nutrient canals had to penetrate dense hyaline CEP before reaching the disc matrix, and then nutrition was received from passive diffusion from CEP (44, 45). Blood-related cells existed in CEP tissue included smooth muscle cells, blood cells and endothelial cells, which provided hard evidence with the previous viewpoint, and also blood vessels in normal CEP are prone to angiogenesis and infiltration, which carried immune cells, thus lead into CEP degeneration. This study then performed cell-cell communication analysis based on different cell type in CEP tissue, to figure out the underlying mechanisms in regulating CEP development. Stromal cells, which produced ECM in CEP, served as development and growth place for different cells, such as chondrocytes, endothelial cells and smooth muscle cells, besides, they also responded to mechanical stimulus. ECM proteins were involved in stem cell maintenance and renewal, which were regarded as pivotal component of stem cell niches (46, 47). The components of niches were intricate and comprised of diverse cell types as well as extracellular factors. Mesenchymal stem/Progenitor cells occupied a specific stem cell niche, which was a special environment where stem cells located in and kept quiescent (48). In CEP tissue of this study, NPMSCs were located in stem cell niche and behaved pivotal roles with different cell populations to regulate the homeostasis of CEP. SPP1/CD44 or CD74/MIF signaling pathways had been reported to regulate bone formation (34, 35), which were demonstrated by our findings that these changes were activated by the interactions between NPMSC and stromal cells. Served as one of the 5 cell surface ligands that functioned primarily in the highly conserved Notch signal pathway, JAG1 was activated by NPMSC cells and acted on other cell populations through JAG1/Notch signaling pathway, which also had essential roles in cancer stem cell differentiation and metastasis, and it was also related with diverse non-tumor diseases including liver, heart, skeletal etc., most were functioned by JAG1/Notch signaling pathway (49, 50). Consequently, the existence of JAG1 in NPMSC cells highlighted the crucial roles of JAG1 as a regulatory molecule in the development of CEP, which may be regarded as a target in developing new diagnostic and therapeutic approaches. Endothelial cells may contribute to angiogenesis by expressing angiogenic factor like VEGF, and they also produced different growth factors like PDGF, and PGF etc., the neovascularization could further bring cytokines as well as immune cells, which finally lead into degeneration of CEP and cause DDD. However, the exact mechanisms should be confirmed by further experiments.

In conclusion, we reported the first combined analysis of WGCNA and scRNA-seq in CEP tissue. These findings revealed the heterogeneity and diverse interactive roles for CEP sub populations during homeostasis and degeneration, indicating potential diagnostic and therapeutic strategies for DDD, and also provided a novel understanding of the potential interactions about CEP. A comprehensive understanding of cell heterogeneity and their key signals within CEP homeostasis may help establish novel insights and strategies of diagnostic and therapeutic targets in DDD.

## Conclusion

We explored and provided a group of gene signatures for researchers to focus on, including BRD4, RAF1, ANGPT1, CHD7 and NOP56, which behaved essential roles in the development of degenerative CEP; then we constructed a transcriptomic landscape of CEP compositions, regulatory and effector chondrocytes had dual roles in maintaining the homeostasis of CEP. Also, NPMSC cells were located in stem cell niche of CEP tissue, and regulated the homeostasis or degeneration of CEP through different cell communications; JAG1/Notch signaling pathway could be served as a novel therapeutic target for degenerative CEP. This is the first conjoint analysis of WGCNA and scRNA-seq in CEP tissue, this study filled the gap in the knowledge of CEP components, further enhanced researchers’ understanding of CEP and their cell niches constitution.

## Data Availability Statement

The datasets presented in this study can be found in online repositories. The names of the repository/repositories and accession number(s) can be found in the article/**Supplementary Material**.

## Author Contributions

Conceived the idea: MY, BG, YW, and WL; Manuscript draft: WL, DW, and YZ; Technical support on analysis: WL; Downloaded and collected data: WL, DW, YZ, and QS; Analyzed the data: WL, SZ, YZ, and ZD; Prepared figures: BG, WL, MY, and YZ; Redressed the manuscript: MY, BG, YW, and WL; Reviewed the manuscript: All authors. This study was completed with teamwork. Each author had made corresponding contribution to the study. All authors contributed to the article and approved the submitted version.

## Funding

This study was supported by grants from the National Natural Science Foundation of China (No. 82072475, 82172475, 81902240), and Efficiency Improvement Project (2019ZTA05-02).

## Conflict of Interest

The authors declare that the research was conducted in the absence of any commercial or financial relationships that could be construed as a potential conflict of interest.

## Publisher’s Note

All claims expressed in this article are solely those of the authors and do not necessarily represent those of their affiliated organizations, or those of the publisher, the editors and the reviewers. Any product that may be evaluated in this article, or claim that may be made by its manufacturer, is not guaranteed or endorsed by the publisher.

## Supplementary Material

Supplementary Table 1 The detailed information of genes in pink module as well as GO, KEGG enrichment functions.

Supplementary Table 2 DEGs among different cell types by scRNA-seq analysis.

Supplementary Table 3 Detailed functional annotations in each cell type.

Supplementary Table 4 Detailed signaling pathways enrichment in each cell type.

Supplementary Figure 1
**(A)**, PCA heatmap showed the expression patterns of the top 30 significantly correlated genes in each component, colors from purple to yellow represented the gene expression levels from low to high, among which PC_1 to PC_10 were displayed. **(B)**, Dot plots showed the top 30 significantly correlated genes in each component, among which PC_1 to PC_4 were displayed.

Supplementary Figure 2 Signaling pathways enrichment analysis for NPMSC, stromal cell, smooth muscle cell, blood cell, endothelial cell, homeostatic chondrocytes, regulatory chondrocytes, and effector chondrocytes, respectively **(A–H)**.

Supplementary Figure 3 The detailed cellular inter-regulatory network between each cell type. Dot size indicated the relative quantity of each cluster; line thickness represented the relative quantity of significant ligand-receptor pairs.
