# Supplementary figures and images for: Revealing the Key MSCs Niches and Pathogenic Genes in Influencing CEP Homeostasis: A Conjoint Analysis of Single-Cell and WGCNA
Source: Front Immunol. 2022 Jun 27;13:933721. doi: 10.3389/fimmu.2022.933721 (PMC9271696; doi:10.3389/fimmu.2022.933721)

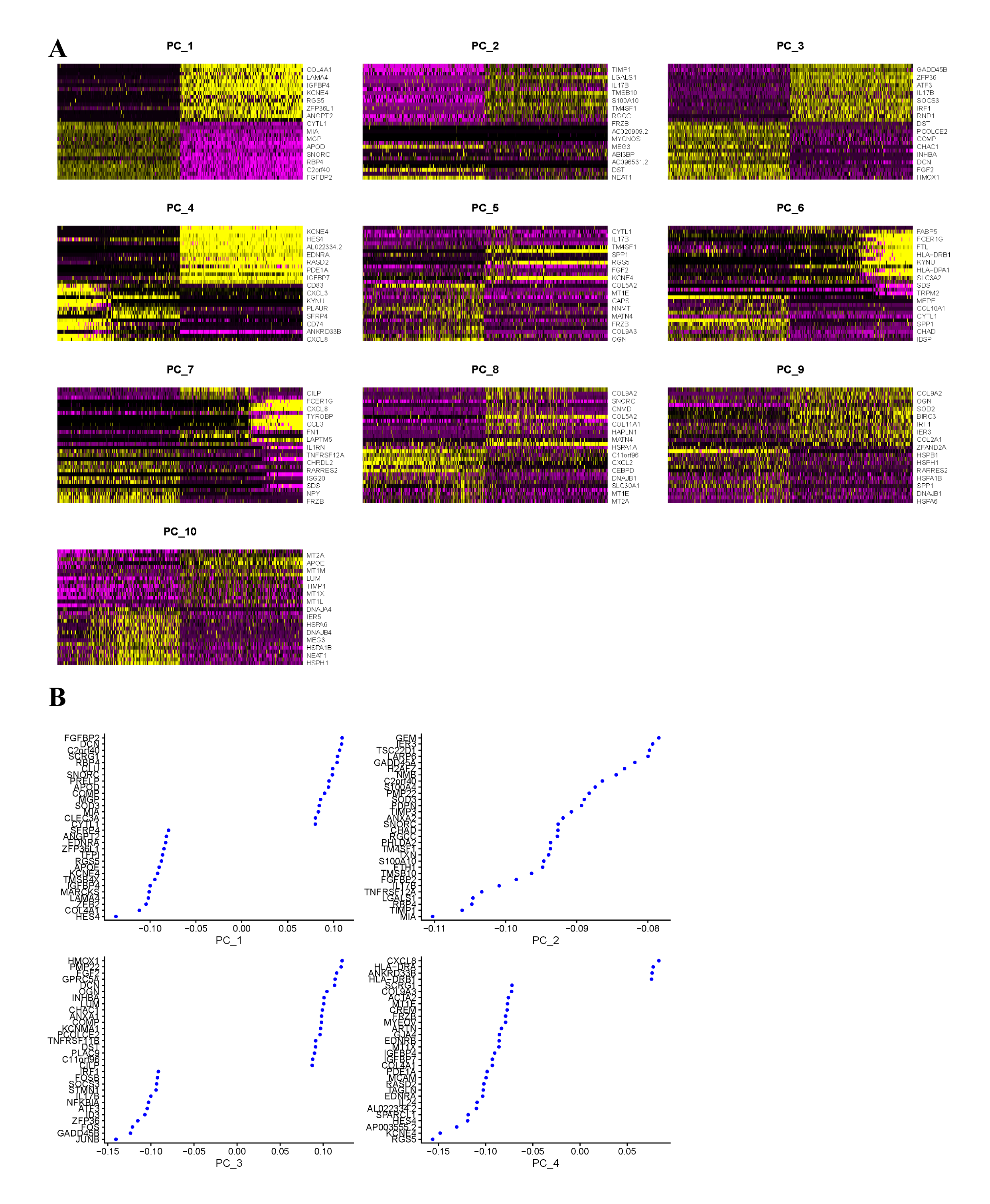

Supplement: Supplementary Figure 1 — (A), PCA heatmap showed the expression patterns of the top 30 significantly correlated genes in each component, colors from purple to yellow represented the gene expression levels from low to high, among which PC_1 to PC_10 were displayed. (B), Dot plots showed the top 30 significantly correlated genes in each component, among which PC_1 to PC_4 were displayed. [file Image_1.tif]

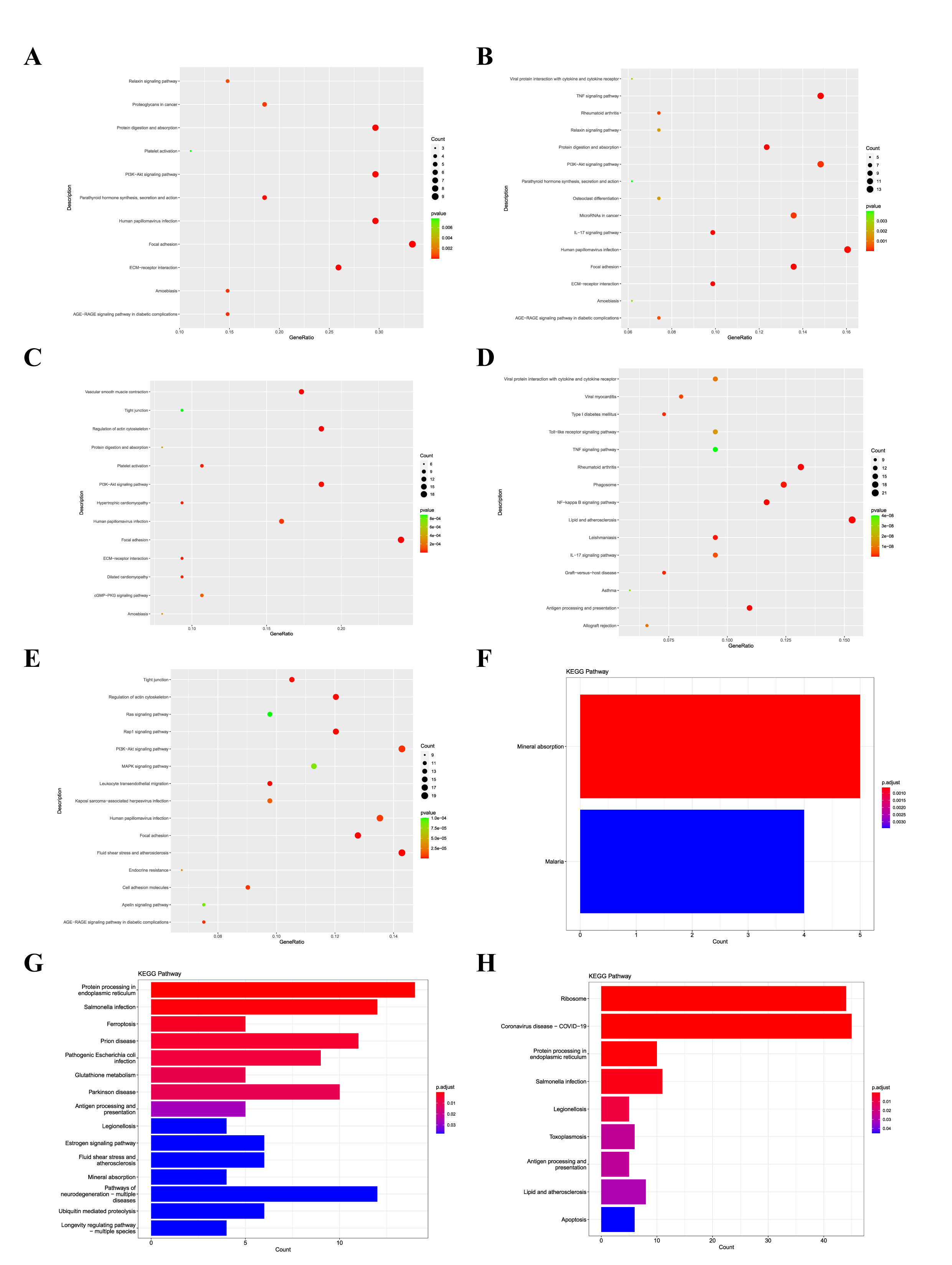

Supplement: Supplementary Figure 2 — Signaling pathways enrichment analysis for NPMSC, stromal cell, smooth muscle cell, blood cell, endothelial cell, homeostatic chondrocytes, regulatory chondrocytes, and effector chondrocytes, respectively (A–H). [file Image_2.tif]

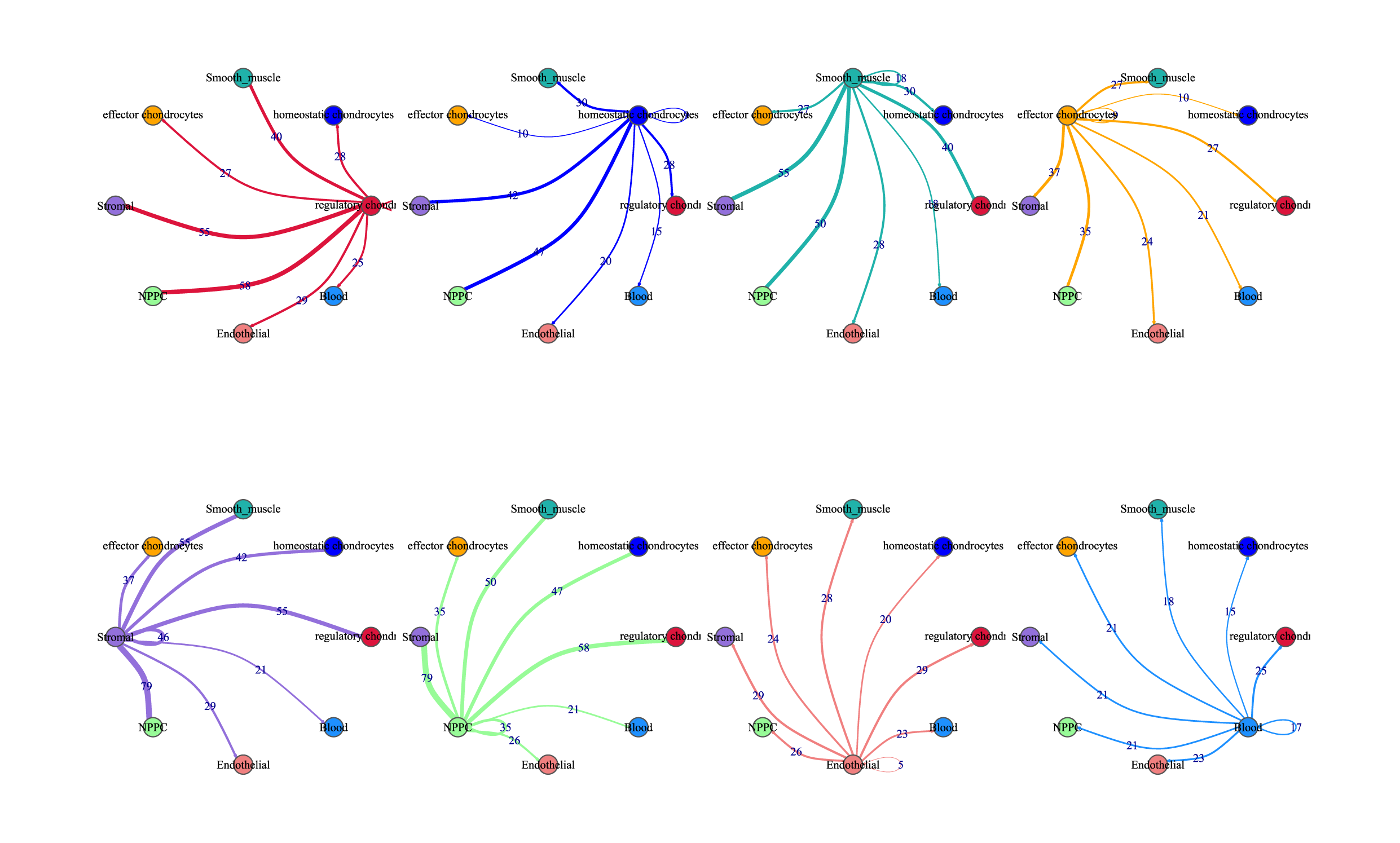

Supplement: Supplementary Figure 3 — The detailed cellular inter-regulatory network between each cell type. Dot size indicated the relative quantity of each cluster; line thickness represented the relative quantity of significant ligand-receptor pairs. [file Image_3.tif]
